# The Habenula in the Link Between ADHD and Mood Disorder

**DOI:** 10.3389/fnbeh.2021.699691

**Published:** 2021-06-24

**Authors:** Young-A Lee, Yukiori Goto

**Affiliations:** ^1^Department of Food Science and Nutrition, Daegu Catholic University, Gyeongsan, South Korea; ^2^Primate Research Institute, Kyoto University, Inuyama, Japan

**Keywords:** neurodevelopmental disorder, depression, animal model, dopamine, serotonin, p-factor

## Abstract

Attention-deficit/hyperactivity disorder (ADHD) is a childhood-onset, neurodevelopmental disorder, whereas major depressive disorder (MDD) is a mood disorder that typically emerges in adulthood. Accumulating evidence suggests that these seemingly unrelated psychiatric disorders, whose symptoms even appear antithetical [e.g., psychomotor retardation in depression vs. hyperactivity (psychomotor acceleration) in ADHD], are in fact associated with each other. Thus, individuals with ADHD exhibit high comorbidity with MDD later in life. Moreover, genetic studies have shown substantial overlaps of susceptibility genes between ADHD and MDD. Here, we propose a novel and testable hypothesis that the habenula, the epithalamic brain region important for the regulation of monoamine transmission, may be involved in both ADHD and MDD. The hypothesis suggests that an initially hypoactive habenula during childhood in individuals with ADHD may undergo compensatory changes during development, priming the habenula to be hyperactive in response to stress exposure and thereby increasing vulnerability to MDD in adulthood. Moreover, we propose a new perspective on habenular deficits in psychiatric disorders that consider the habenula a neural substrate that could explain multiple psychiatric disorders.

## Introduction

The current diagnostic manuals of psychiatric disorders, such as the Diagnostic and Statistical Manual of Mental Disorders, Fifth Edition (DSM-5) (Americal Psychiatric Association [APA], 2013) and International Classification of Diseases, 11th Edition (ICD-11) (World Health Organization [WHO], 2018), classify psychiatric disorders into categories as distinct entities. However, patients who have one category of a psychiatric disorder are often diagnosed with other comorbid disorders in other categories. Such observations raise the possibility that psychiatric disorders may be dimensional rather than categorical. Thus, a few or perhaps a single factor, such as a general psychopathological factor, i.e., the p-factor, may explain all psychiatric conditions (Wright et al., 2013; Caspi et al., 2014; Kotov et al., 2017; Caspi and Moffitt, 2018).

Attention-deficit/hyperactivity disorder (ADHD) is a childhood-onset neurodevelopmental disorder (Biederman and Faraone, 2005). In contrast, major depressive disorder (MDD) is a mood disorder that typically emerges in adulthood (Kupfer et al., 2012). Accumulating evidence suggests that both disorders may involve deficits in the habenula. The habenula is a set of epithalamic nuclei consisting of the medial and lateral parts that receive inputs and integrate information from limbic structures and the basal ganglia. The habenula in turn sends outputs to midbrain nuclei where dopamine (DA) and serotonin (5-HT) neurons are located (Hikosaka et al., 2008; Boulos et al., 2017; Fakhoury, 2017; Hu et al., 2020). Relationships between ADHD and MDD, which are distinct categories of disorders and seemingly unrelated to each other, would be worth considering in relation to the roles of the habenula in the regulation of mesocorticolimbic DA and 5-HT transmission in the context of a dimensional model.

In this article, we first briefly summarize the literature demonstrating correlations between ADHD and MDD, followed by a discussion of the effects of habenular deficits in these disorders based primarily on animal models. Then, we further propose that investigations examining habenular dysfunction not in the context of a unitary, categorized disorder but as a common factor underlying multiple psychiatric conditions would be a thriving future direction.

## ADHD and MDD Comorbidity

ADHD comprises the core symptoms of hyperactivity, impulsivity, and attention deficit that is classified into three types, depending on which symptoms are prominent: inattentive, hyperactive/impulsive, and combined types (Biederman and Faraone, 2005). MDD is a mood disorder involving depressed mood and loss of pleasure and interest (Kupfer et al., 2012). MDD typically occurs in adulthood, although nowadays a significant number of children and adolescents are also diagnosed with MDD (Luby, 2009; Maughan et al., 2013).

Some MDD symptoms could be antithetical to those of ADHD. For instance, psychomotor retardation (Bennabi et al., 2013) in MDD could be considered the opposite of hyperactivity as psychomotor acceleration in ADHD. Rumination (Figueroa et al., 2019) is the focused and persistent thoughts of negative content causing emotional distress, whereas ADHD subjects exhibit excessive spontaneous mind wandering (Bozhilova et al., 2018). Moreover, abnormally augmented behavioral inhibition has been reported as a risk factor for MDD (Kasch et al., 2002; Gladstone and Parker, 2006). In contrast, impulsivity is a core symptom of ADHD.

There are other interesting coincidences between ADHD and MDD. Both disorders involve sleep disturbances, such as insomnia (Konofal et al., 2010; Hvolby, 2015; Pandi-Perumal et al., 2020), although hypersomnia is also often observed in MDD (Lopez et al., 2017). Circadian rhythms are also compromised in ADHD (Kooij and Bijlenga, 2013; Wynchank et al., 2016; Lunsford-Avery and Kollins, 2018). In MDD, symptom severity fluctuates within a day and even across seasons, with more severe symptoms in the winter (Germain and Kupfer, 2008; Boyce and Barriball, 2010). Numerous studies have demonstrated that the habenula plays critical roles in the regulation of both sleep and circadian rhythms (Valjakka et al., 1998; Salaberry and Mendoza, 2015; Bano-Otalora and Piggins, 2017; Mendoza, 2017; Aizawa and Zhu, 2019).

Subjects with ADHD are frequently diagnosed with other comorbid disorders, such as autism spectrum disorder, mood and anxiety disorder, drug addiction, and personality disorder (Katzman et al., 2017; Gnanavel et al., 2019). Epidemiological surveys in the United States have reported that, although the prevalence of MDD in typically developing children is only approximately 1%, it approaches approximately 15% among children with ADHD (Larson et al., 2011). The prevalence of MDD in adult ADHD subjects is twice as high (19%) as that in subjects without ADHD (8%) (Kessler et al., 2006). Longitudinal and meta-analysis studies have also demonstrated that childhood ADHD increases the risk of MDD during adolescence and young adulthood with an odds ratio of approximately 1.2–1.3 (Meinzer et al., 2014; Bron et al., 2016; Riglin et al., 2020).

Various mediators have been suggested regarding the comorbidity of ADHD and MDD. These include psychosocial factors, such as parent management (Ostrander and Herman, 2006), peer problems (Powell et al., 2020), academic attainment (Powell et al., 2020), emotion regulation (Seymour et al., 2012), anxiety (Roy et al., 2014), and disruptive behaviors (Roy et al., 2014). Neuroimaging studies have also reported neuronal mediators, such as decreased left hippocampal volume, and impairments in intrinsic functional connectivity between the hippocampus and orbitofrontal cortex (Posner et al., 2014) and between the anterior cingulate cortex and dorsolateral prefrontal cortex (PFC) (Whitfield-Gabrieli et al., 2020).

## Genetic Correlations Between ADHD and MDD

Recent genetic studies have substantiated associations between ADHD and MDD. In a genome-wide association study (GWAS) with ADHD subjects, Ebejer et al. (2013) identified the strongest association with the gene for GPR139. GPR139 is an orphan G-protein coupled receptor whose role has been suggested to be a sensor of L-tryptophan and L-phenylalanine (Liu et al., 2015; Vedel et al., 2020). GPR139 has also been suggested to be relevant in MDD (Vedel et al., 2020). GPR139 signaling in the habenula was recently found to play an important role in fear learning in zebrafish (Roy et al., 2021).

Direct evidence of the associations between ADHD and MDD comes from GWAS meta-analyses examining genetic correlations with several different psychiatric disorders. A GWAS meta-analysis with ADHD subjects by Demontis et al. identified 12 genome-wide significant loci that were modestly, but significantly, correlated with depressive symptoms and MDD (r_*g*_ = 0.42) (Demontis et al., 2019). A similar meta-analysis of GWASs with an even larger sample size of MDD patients by Wray et al. identified 44 loci that were correlated with ADHD at a similar strength (r_*g*_ = 0.42) (Wray et al., 2018). Thus, similar strengths in the associations between ADHD and MDD have been observed in analyses using different cohorts, suggesting that the association between these disorders is highly consistent. Two GWAS meta-analyses by the Cross-Disorder Group of the Psychiatric Genomics Consortium that examined associations in five and eight psychiatric disorders have demonstrated genetic correlations of similar strengths between ADHD and MDD to those reported in other meta-analysis studies, along with identification of associations of single nucleotide polymorphisms (SNPs) on chromosomes 3p21 and 10q24 and CACNB2, the gene encoding a voltage-gated L-type calcium channel, suggesting that L-type calcium channels could be a candidate molecule linking MDD and ADHD (Cross-Disorder Group of the Psychiatric Genomics Consortium, 2013, 2019). A GWAS meta-analysis by Powell et al. also compared ADHD and MDD, identifying 14 SNPs with concordant directions of effect for both disorders, with the estimated genetic correlation being r_*g*_ = 0.52 (Powell et al., 2021).

Collectively, these GWASs have demonstrated modest, but highly consistent, SNP-based genetic correlations between ADHD and MDD, consolidating the associations between these disorders.

## Habenula in ADHD and MDD

Extensive research has been conducted to reveal the molecular and cellular mechanisms in the habenula that influence cognitive and affective functions as well as dysfunction implicated in psychiatric disorders. There are many comprehensive reviews that have summarized these studies (Hikosaka et al., 2008; Boulos et al., 2017; Fakhoury, 2017; Hu et al., 2020), such that here we refer only briefly to some findings about habenular deficits relevant to the pathophysiology of MDD and ADHD.

Using animal models of MDD, hyperactivity in the lateral nucleus of the habenula has consistently been demonstrated (Browne et al., 2018; Yang et al., 2018; Aizawa and Zhu, 2019; Gold and Kadriu, 2019). Specific patterns of acute and chronic electrical stimulation (Li et al., 2011; Meng et al., 2011; Tchenio et al., 2017) or pharmacological inhibition (Winter et al., 2011) of the habenula attenuate MDD-like behaviors in animal models. This is further supported by a recent case report study showing improvement of symptoms with deep brain stimulation of the habenula in treatment-resistant MDD patients (Wang et al., 2020). In contrast, human neuroimaging and postmortem studies have been inconsistent. Some studies have reported larger, and others found smaller, volumes of the habenula in MDD patients than in healthy subjects (Ranft et al., 2010; Savitz et al., 2011; Carceller-Sindreu et al., 2015; Schmidt et al., 2017). Functional imaging studies have demonstrated higher or lower than normal habenular activation in MDD patients (Roiser et al., 2009; Furman and Gotlib, 2016; Lawson et al., 2017). Such inconsistent findings are not surprising, given the heterogeneous nature of symptoms across MDD patients (Kupfer et al., 2012). Thus, there are huge gaps between animal model studies and the realm of human psychiatric conditions, and findings with animal models are unlikely to be directly translatable into human situations (Lee and Goto, 2013; Planchez et al., 2019; Baker et al., 2020; Stanford, 2020).

Compared with MDD and other psychiatric disorders, such as schizophrenia and drug addiction (Lecourtier and Kelly, 2005; Heldt and Ressler, 2006; Lecourtier et al., 2006; Velasquez et al., 2014; Boulos et al., 2017; Fakhoury, 2017; Mathuru, 2018; Li et al., 2019; Mathis and Kenny, 2019; Hu et al., 2020), research examining habenular deficits in ADHD pathophysiology are scarce. When we investigated the effects of neonatal habenular lesions (NHLs) in rats, there were unexpected findings with NHLs causing an assortment of behavioral alterations resembling ADHD symptoms. Thus, rats with NHLs exhibit spontaneous hyperlocomotion, more impulsive choices in decision-making tasks, and shorter attention spans in object exploration, all of which were ameliorated by amphetamine (Lee and Goto, 2011). Moreover, these behavioral alterations dynamically changed through development, with hyperlocomotion and impulsivity apparent only in childhood, whereas attention deficits persisted up until adulthood. Such developmental patterns are consistent with the waxing and waning of ADHD symptoms over development (Biederman and Faraone, 2005; Spencer et al., 2007). This novel aspect of the NHL model distinguished it from other conventional animal models of ADHD, such as spontaneously hypertensive rats (Russell, 2011).

NHLs also cause an assortment of neural alterations, such as a smaller PFC volume (Lee and Goto, 2011) and abnormally augmented amygdala–PFC connectivity (Kim et al., 2021), which are also consistent with those found in ADHD individuals (Plessen et al., 2006; Shaw et al., 2007; Batty et al., 2010; Posner et al., 2011; Batty et al., 2015; Van Dessel et al., 2018). We further found that tissue concentrations of DA and 5-HT were balanced in mesocorticolimbic regions of normal rats, but these levels were disrupted in NHL rats, suggesting that imbalances between DA and 5-HT may be more important than alterations in DA or 5-HT levels alone in NHL-induced behavioral alterations (Lee et al., 2021). There has been only one human neuroimaging study that investigated habenular deficits in ADHD subjects to date (Arfuso et al., 2019). This study demonstrated that intrinsic functional connectivity between the habenula and putamen was impaired in ADHD subjects. Additional human studies are needed to identify habenular deficits in ADHD.

Although a neonatal “lesion” gives an impression of damage in the habenula, NHLs result in smaller nuclear sizes of both medial and lateral habenula than those of normal rats, which could be due to the manipulation during early brain development. This raises a couple of issues to be further examined. First, since both medial and lateral nuclei of the habenula are affected by NHL, it has remained elusive whether and in what way neonatal manipulations of either the medial or lateral nucleus alone would yield distinct alterations. For instance, ADHD symptoms are grouped into hyperactive-impulsive dominant, inattention dominant, and mixed types (Biederman and Faraone, 2005). NHLs affecting both the medial and lateral nuclei induce behavioral alterations consistent with the mixed types. Thus, a selective neonatal lesion to either the medial or lateral nucleus may induce hyperactive-impulsive or inattention dominant types of alterations. Another issue is whether animals with smaller habenular nuclei as naturally occurring individual variations may also exhibit more hyperactive, impulsive, and inattentive traits than those of larger habenular nuclei. On the other hand, smaller habenular nuclei caused by NHL may produce a condition equivalent to the hypoactive state of the habenula, such that volume size itself may not be the important factor. This is supported by inconsistent findings regarding anatomical and functional habenular changes in MDD patients (Roiser et al., 2009; Ranft et al., 2010; Savitz et al., 2011; Carceller-Sindreu et al., 2015; Furman and Gotlib, 2016; Lawson et al., 2017; Schmidt et al., 2017).

**FIGURE 1:**
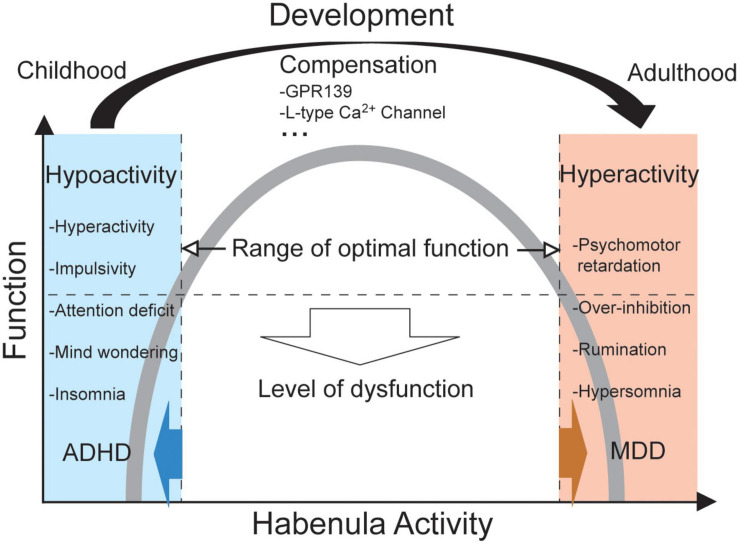
A diagram illustrating the hypothesis. In terms of habenular function in relation to its activity (i.e., an inverted U-shaped relationship), ADHD and MDD may be antithetical. A dysfunctional level of hypoactivity in the habenula may induce an assortment of symptoms relevant to ADHD, whereas hyperactivity of the habenula may cause those relevant to MDD. A transition from hypoactivity to hyperactivity, but not vice versa, may take place over the course of brain development from childhood to adulthood, as compensation. Such a process may involve several molecules, such as GPR139 and L-type calcium channels.

Taken together, a hypothesis has emerged that may explain the pathophysiology of comorbid ADHD and MDD (Figure 1). In particular, hypoactivity of the habenula early in development may initially produce ADHD-like behaviors with molecular alterations, such as differences in GPR139 and L-type calcium channels as suggested in GWASs. As the brain develops and matures into adulthood, such hypoactive habenula undergoes compensatory changes that subsequently increases vulnerability to MDD by priming the habenula for hyperactivation on exposure to stress.

## Habenula as a Neuronal p-Factor?

In addition to ADHD and MDD, habenular deficits have been implicated in other psychiatric disorders. As is the case with MDD, although a relatively large number of animal model studies have provided support for habenular deficits in schizophrenia (Lecourtier and Kelly, 2005; Heldt and Ressler, 2006; Lecourtier et al., 2006; Boulos et al., 2017; Fakhoury, 2017; Li et al., 2019; Hu et al., 2020) and drug addiction (Velasquez et al., 2014; Boulos et al., 2017; Fakhoury, 2017; Mathuru, 2018; Mathis and Kenny, 2019; Hu et al., 2020), there has been little or inconsistent evidence regarding habenular deficits in human patients with these disorders (Shepard et al., 2006; Ranft et al., 2010; Boulos et al., 2017; Fakhoury, 2017; Zhang et al., 2017; Schafer et al., 2018; Germann et al., 2020; Hu et al., 2020).

Implications of habenular deficits in such an assortment of psychiatric disorders are not surprising, given the function of the habenula (Hikosaka et al., 2008). Thus, the lateral habenular nucleus regulates DA and 5-HT neuron activities in the midbrain nuclei directly and indirectly, respectively, through the rostrotegmental nucleus. Moreover, the medial habenular nucleus regulates the DA and 5-HT systems indirectly through the interpeduncular nucleus. DA and 5-HT are in turn neurochemical substances whose alterations are implicated in most, if not all, psychiatric disorders (Esposito et al., 2008). However, such a notion raises concern about how habenular deficits should be considered in the categorical model of psychiatric disorders.

A current diagnosis of psychiatric disorders is based on the categorical model (Americal Psychiatric Association [APA], 2013; World Health Organization [WHO], 2018). In this model, each disorder has unique symptoms and causes that are independent from other disorders. Accordingly, most studies investigating the neural mechanisms of psychiatric disorders, including those related to habenular deficits, follow this model and attempt to elucidate a pattern of deficits unique to a single psychiatric disorder. Such a categorical model does not comply with the idea that deficits of a single brain area, such as the habenula, could be involved in multiple disorders.

As an alternative to the categorical model, the dimensional model has been considered, especially in childhood psychiatry (Achenbach and Edelbrock, 1981; Sourander and Helstela, 2005; Wright et al., 2013; Willner et al., 2016; Kotov et al., 2017). In this model, two factors, internalizing and externalizing dimensions, are considered to underlie different psychiatric disorders. The internalizing dimension explains anxious and depressive symptoms, whereas the externalizing dimension explains aggressive, antisocial, and hyperactive-impulsive symptoms. It is interesting to note that the symptoms in the internalizing and externalizing dimensions are often discussed in relation to the functions of 5-HT and DA transmission, respectively. Thus, although this is highly speculative, DA/5-HT imbalance may explain internalizing and externalizing dimensions (e.g., imbalance toward DA- and 5-HT-predominant conditions lead to externalizing and internalizing symptoms, respectively).

Although it has been suggested that patients are more likely to have comorbidities of psychiatric disorders within the same dimension, correlations have also been observed between externalizing and internalizing symptoms (Wright et al., 2013; Willner et al., 2016), which corresponds to the relationship between ADHD and MDD, as ADHD is related to the externalizing dimension, whereas MDD is related to the internalizing dimension. Thus, a one-step higher and more generalized factor that is inclusive of both internalizing and externalizing dimensions may be required to explain the ADHD and MDD relationships. Such a latent factor has recently been proposed that is mutually involved in the diagnoses of all the different psychiatric disorders, which is denoted as a general psychopathological factor or p-factor (Caspi et al., 2014; Caspi and Moffitt, 2018). The presence of the p-factor is supported by the number of studies, including investigations that have demonstrated substantial genetic overlaps between different psychiatric disorders (Cross-Disorder Group of the Psychiatric Genomics Consortium, 2013, 2019; Golovina et al., 2020). The habenula, which links externalizing and internalizing dimensions, along with its involvement in other psychiatric disorders, could therefore be a promising candidate for a neuronal substrate of the p-factor.

Future investigations that clarify the impacts of habenula deficits in psychiatric disorders may be fertile in the context of general psychopathological factors, such as how habenular deficits can explain the comorbidity of multiple disorders, rather than being associated with deficits in a particular psychiatric disorder.

## Conclusion

We have proposed a hypothesis that habenular hypoactivity early in development may produce ADHD-like behaviors. The habenula may subsequently go through compensatory changes across development that leads to hyperactivity with an increased vulnerability to stress and MDD. Thus, the habenula may be a crucial brain region linking ADHD and MDD. Moreover, the roles of the habenula could be generalized across multiple psychiatric disorders beyond ADHD and MDD as a neural substrate of the p-factor.

## Data Availability Statement

The original contributions presented in the study are included in the article/supplementary material, further inquiries can be directed to the corresponding author/s.

## Author Contributions

YAL and YG conceived the idea and wrote the article. Both authors contributed to the article and approved the submitted version.

## Conflict of Interest

The authors declare that the research was conducted in the absence of any commercial or financial relationships that could be construed as a potential conflict of interest.
